# Trans-cardiac perfusion of neonatal mice and immunofluorescence of the whole body as a method to study nervous system development

**DOI:** 10.1371/journal.pone.0275780

**Published:** 2022-10-13

**Authors:** Andrea Pérez Arévalo, Anne-Kathrin Lutz, Ekaterina Atanasova, Tobias M. Boeckers

**Affiliations:** 1 Institute for Anatomy and Cell Biology, Ulm University, Ulm, Germany; 2 Deutsches Zentrum für Neurodegenerative Erkrankungen (DZNE), Ulm Site, Ulm, Germany; Technion Israel Institute of Technology, ISRAEL

## Abstract

Whole animal perfusion is a well-established method that has been used for the past decades in multiple research fields. Particularly, it has been very important for the study of the brain. The rapid and uniform fixation of tissue is essential for the preservation of its integrity and the study of complex structures. For small tissue pieces submerging in formaldehyde solution oftentimes is sufficient to get a good fixation, larger tissues or organs with a more complicated structure present a greater difficulty. Here, we report the precise parameters to successfully perform trans-cardiac perfusion of neonatal mouse pups that allows a uniform fixation of the whole body for subsequent structural analysis and immunohistochemistry. In comparison to standard perfusion procedures of adult mice, changes in the pump velocity, the buffer volume and in the needle size lead to high quality fixation of neonatal mice pups. Further, we present a whole-body section staining, which results in a highly specific immunofluorescence signal suited for detailed analysis of multiple tissues or systems at the same time. Thus, our protocol provides a reproducible and reliable method for neonatal perfusion and staining that can rapidly be applied in any laboratory. It allows a high quality analysis of cellular structures and expression profiles at early developmental stages.

## Introduction

The brain is a highly complex structure consisting of various cell types [1, 2]. The adult rodent brain has been extensively studied for the last few decades, particularly focusing on understanding the structural and functional connection between neurons and glial cells. Nevertheless, the knowledge on how these connections are formed during development is still limited.

To study the structure and expression patterns of the brain and its different cell types requires the preservation of the integrity of the tissue. To admire this, many methods are based on trans-cardiac perfusion of animals with formaldehyde solution [3]. In this method, the fixative solution is introduced through the left ventricle of the heart to the vascular system and reaches all the cells via the circulatory system and the capillary net [3]. There is also the possibility of fixating the tissue via immersion [4]. However, the effectiveness of this method is limited, depending on the size of the specimen, the fixative does not reach the inner cell layers [4]. In addition, the fixation by perfusion provides faster preservation than the fixation by immersion.

The perfusion of adult mice and other rodents has been long established [3], serving as a standard method for multiple structural and biochemical analysis. However, the conduction of the same method in neonatal rodents, or during their early development, is not well described in literature. In neonatal pups the circulatory system is harder to reach, to manipulate and to use for perfusion. This entails one of the main problems when perfusing such small animals. The perfusion of neonatal pups has been reported in the literature before, however, in the methods section the specific protocol is usually not specified, making it hard to have a reliable result [5].

Moreover, a reliable method to study multiple tissues in one section during developmental stages is lacking in literature when investigating for example the interaction between multiple tissues, like the gut-brain axis [6–8]. In this respect, our protocol maintains the original structure of the complete neonatal mouse pup, allowing the staining of multiple tissues in the same slide. Thus, resources are saved and researchers have a fast procedure on hand that provides them with valuable tissue leading to high quality immunohistochemical stainings that can help dissect tissue interactions and their role in neural development. In this study, we provide the exact settings and a detailed protocol on how to successfully perform trans-cardiac perfusion in neonatal mice followed by whole body immunohistochemical stainings.

## Materials and methods

The protocol described in this peer-reviewed article is published on protocols.io, dx.doi.org/10.17504/protocols.io.bp2l61ow5vqe/v1 and is included for printing as S1 File with this article.

### Fixation by immersion

Neonatal mice pups were anesthetized using a mix of Xylazin and Ketamine (520mg/Kg ketamine and 78mg/Kg Xylacine) in a saline solution. 20uL of this mix were injected into the intraperitoneal area using a small insulin syringe. Once we checked the heartbeat and the breathing stopped, the paw reflex was checked. A small incision was made in the thorax of the pups leaving the organs exposed. After, pups were immersed in a 4% w/v PFA solution, and left for 24 hours at 4°C. The day after, pups were washed with PBS-/- and immersed in a gradient of sucrose (10%, 20%, 30%) and left until they dropped to the bottom of the falcon. After that, freezing and cryosectioning of the samples were performed following the protocol mentioned previously.

### Animals

All animal experiments were performed in compliance with the guidelines for the welfare of experimental animals issued by the Federal Government of Germany and approved by the Regierungspraesidium Tübingen and the local ethics committee at Ulm University (ID Number: O.103). C57BL/6 mice were used for breeding. They were housed under constant temperature (22 ± 1°C) and humidity (50%) conditions with a 12 h light/dark cycle and provided with food and water *ad libitum*.

## Expected results

**Fig 1:**
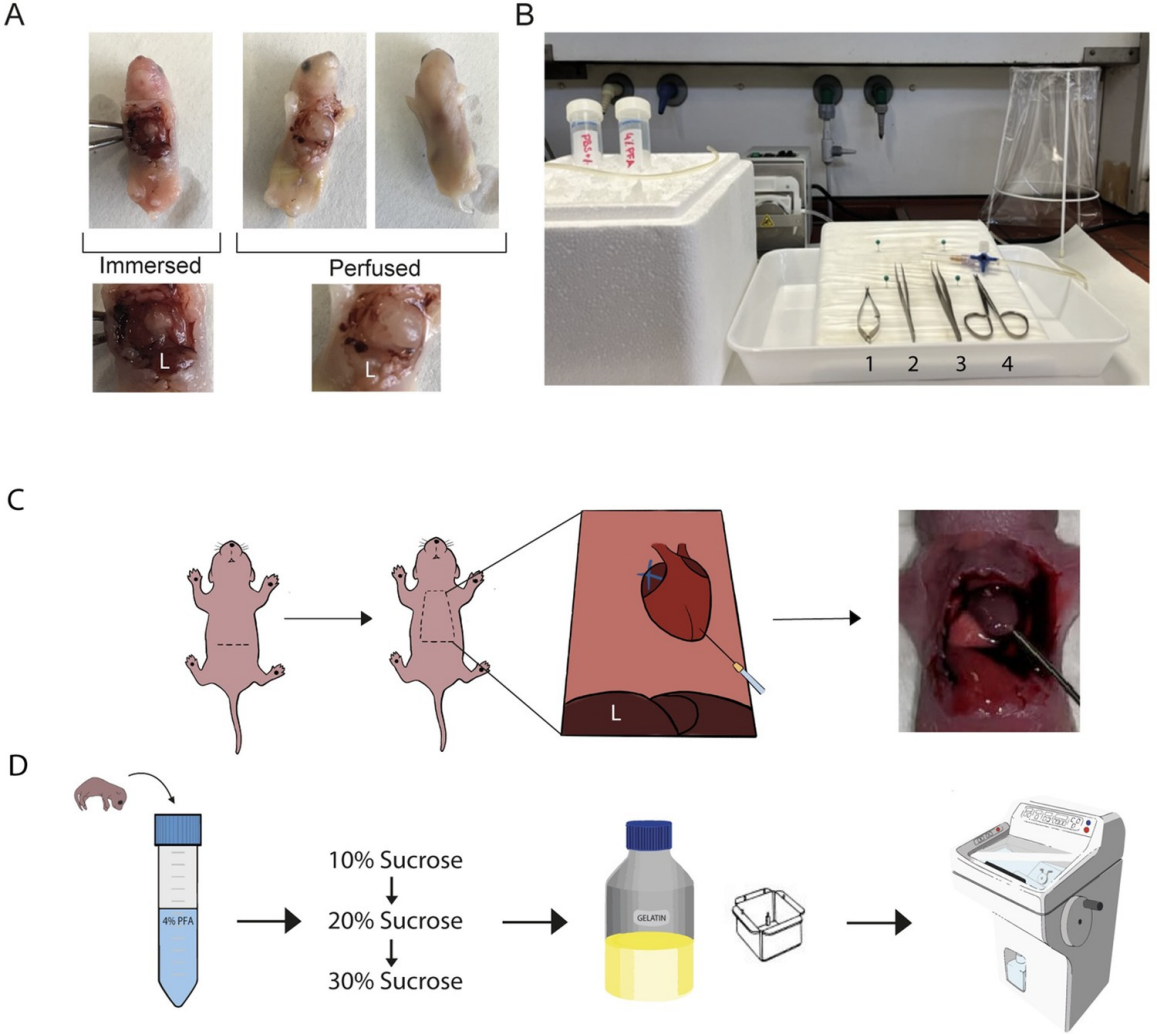
Schematic view of the protocol. a. Comparison between fixation by immersion and trans-cardiac perfusion in neonatal mice. The liver (L) is shown in detail. b. Set-up for the perfusion with the materials needed (1. Microscissors, 2. Straight Forceps, 3. Curved Forceps, 4. Small Scissors). c. Scheme of the surgical procedure during the perfusion. d. Scheme of the post-fixation procedure to prepare the tissue for cryosectioning.

Analysis of neonatal mouse pups in immunohistochemical stainings is an essential tool to study the development and maturation of cells and organs. An easy approach is to submerge the whole pup into formaldehyde solution and to proceed from there following standard procedures [4]. However, by this the tissue is not cleared from blood as seen from the pale color of the skin (Fig 1A, left panel) in comparison to well-cleared perfused tissue (Fig 1A, right panel).

The liver is a good indicator of the clearance by turning pale (Fig 1A, magnification). In addition to this, perfusion offers several advantages (Table 1). The whole body can be cut and stained in one, while one tissue at a time is usually fixed when using immersion. The quality of the staining improves significantly, cryo-sectioning of the whole pup becomes possible since the skin is not sticking to the cryo-knife, the whole body gets fixed equally through the distribution of the formaldehyde solution through the circulatory system and the procedure is faster than perfusion by immersion.

**Table 1:** Comparison between fixation by immersion and trans-cardiac perfusion.

	Fixation by immersion	Trans-cardiac perfusion
*Blood clearing*	No	Yes
*Fixation of multiple tissues*	Yes (separate tissues)	Yes, maintaining complete structure
*Fixative penetration*	Superficial	Total
*Time*	24-48h	20min + Post-fixation (1h-12h)
*Ease of cutting (Whole Body)*	Difficult (only superficial fixation of the body)	Good

The setup for the perfusion is displayed in Fig 1B. After anesthesia, the chest is opened as depicted in Fig 1C. Essentially, the right atrium of the heart has to be cut as indicated with the blue cross, to allow the fixative to exit the body. Furthermore, a size 27G needle has to be used for the perfusion, since we found that larger needles cannot reliably be introduced into the mouse heart, leading to eventual failure of the perfusion. Further, using a 27G needle makes it easier, compared to the ones usually used on adults, to control the speed and volume of the buffers that are introduced into the circulatory system and allows them to follow a constant flow. Before introducing the formaldehyde solution in the circulatory system, the blood has to be cleared out with a saline solution, in this case PBS-/-, so the fixative solution can reach all the tissues equally. As with other fixation methods [9], the pH and osmolality of the solution has to be adjusted. The pH has to match the pH of the blood to keep the tissue as ideal as possible and not cause acidosis or alkalosis, which could damage the tissues of interest [9]. In addition, the velocity and the pressure of the pump as well as the fixation time have to be adjusted to avoid the rupture of any vessel and consequently the failure of the procedure and loss of the sample. We tried different velocities and ended up using a velocity of 1mL/min to compromise the pressure the tissue can stand, the lowest velocity the pump is able to keep without standing still and the time the perfusion takes. To make sure that we cleared all the blood out we used 10mL of PBS-/-. Afterwards, we used the same volume of formaldehyde solution to fix all the tissues. Counting both parts, we ended up with a total fixation time of 20min (as shown in Table 2). Perfusion is completed, as soon as the color of the liver has changed from red to a pinkish appearance.

**Table 2:** Main differences between the adult and the neonatal trans-cardiac perfusion protocol.

	Adult Trans-cardiac perfusion	Neonatal Trans-cardiac perfusion
*Needle size*	26G	27G
*pH Solutions*	7.4	7.4
*PBS-/- Volume used*	45mL	10mL
*PFA Volume used*	45mL	10mL
*Pump Velocity*	2.5ml/min	1ml/min
*Total time*	~ 40min	20 min
*Post-fixation*	Brain → overnight	Whole body → overnight Brain → 2h

After perfusion, we submerge the whole pup in formaldehyde solution followed by a gradual dehydration in sucrose solution (Fig 1D). The pup was then embedded in gelatin (see S1 File for details). We found that gelatin works significantly better than other embedding solutions for cryo-sectioning as for example Tissue Tek O.C.T. (O.C.T.), since the gelatin sticks well to the skin of the pup and therefore prevents the skin from separating from the rest of the tissue while cutting later for immunohistochemistry [10].

**Fig 2:**
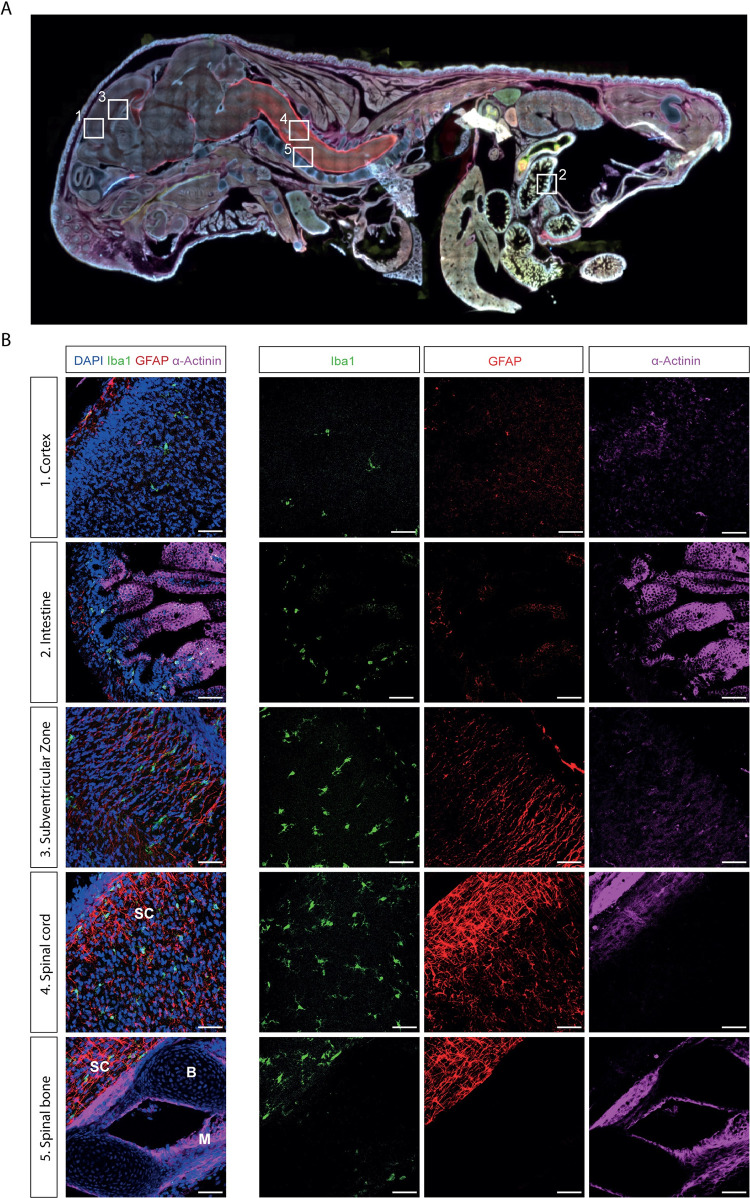
Immunohistochemistry of neonatal perfused mouse tissue. a. Whole body staining (magnification: 10x) (blue: DAPI, red: GFAP, green: Iba1, magenta: ɑ-Actinin) b. Magnified (magnification: 30x) images of different regions of the previous staining (1. Cortex, 2. Intestine, 3. Subventricular Zone, 4. Spinal cord, 5. Spine) (blue: DAPI, red: GFAP, green: Iba1, magenta: α-Actinin). Scale bar: 500μm. SC: Spinal cord, B: bone, M: muscle.

To demonstrate immunofluorescent staining using this perfusion method, we used antibodies directed against Ionized Calcium-Binding Adapter Molecule 1 (Iba1), expressed in macrophages and microglia [11–13], Glial Fibrillary Acidic Protein, expressed in astrocytes and Schwann cells (GFAP) [14], α-Actinin (a protein of the muscle sarcomere) [15, 16], and counterstained the section with DAPI. The overview in Fig 2A shows that all organs of the pup are stained equally well. The tissues stayed in place and were neither disrupted nor were the organs pulled apart. The skin was still surrounding the whole section. This method is suited to both assess different organs at the same time, as shown in the overview (Fig 2A), but also to perform detailed acquisition of high-quality tissue, as shown in the magnifications (Fig 2B).

The regions shown in Fig 2B were chosen to display that the cellular organization of different tissues of interest, such as the brain, spinal cord and intestine, is well maintained after perfusion and immunocytochemistry. The advantage of staining the whole body in one section, and having multiple tissues on the same slide as shown in Fig 2A, allows for the study of the interaction of various tissues or organs during development. For example, it has recently been described that the gut impacts the brain during development [6–8]. To show the advantage of this technique to be used for this type of studies we decided to show stainings from the nervous system, including the brain cortex (Fig 2B-1) along with a staining from the digestive system, like the intestines (Fig 2B-2). Iba1 is expressed in macrophages and microglia [11, 12], GFAP is expressed in multiple glial cells, including astrocytes [14]. Although Iba1 positive cells are distributed throughout the body (Fig 2A), in the brain Iba1 positive microglia specially localize in the subventricular zone (Fig 2B-3), and their distribution becomes more dispersed towards the cortex (Fig 2B-1), as published previously [17]. In the intestine (Fig 2B-2) the distribution of Iba1 positive macrophages in the muscularis externa of the intestine [18], and the α-Actinin positive muscle cells covering the villus can be observed. The distribution of astrocytes in the spinal cord (Fig 2B-4) reflects the distinction between gray matter and white matter [19, 20] with more astrocytes being expressed in white matter. In addition, α-Actinin expressing muscle cells are visible. This method also allows for the cutting and staining of bone tissue (Fig 2B-5). Next to the GFAP-positive spinal cord and the α-Actinin-positive muscle cells the spinal bones are stained with DAPI (Fig 2B-5).

## Discussion

We demonstrate that trans-cardiac perfusion of the neonatal mouse offers a fast and reliable way to obtain tissue for quality immunofluorescent staining. While there are other possibilities to fix tissues, they don´t provide a complete fixation and excellent preservation of multiple organs and structures at the same time. The benefits of this approach as compared to others that have been used until now [4], are that we are able to get rid of the blood in the circulatory system, and thus, it can be used for the fixation of the different organs, providing more stable and compact tissue. Moreover, the complete fixation of the body provides an easier manipulation while cutting, since it also fixes the skin and connective tissue, which are the parts of the body that can pose a bigger difficulty during the complete body sectioning. Another advantage of this protocol is that you can have the tissue ready for immunohistochemistry in a short period. While perfusion by submersion can take between 24–48 hours, the trans-cardiac perfusion roughly takes 20 minutes plus the post-fixation time, which can vary from 1 hour (if you have only one tissue, e.g. brain) to 12 hours (if you have the whole body). Therefore, trans-cardiac perfusion of neonatal pups helps to advance and broaden the knowledge of early developmental stages of the mouse.

## Supporting information

S1 File Step-by-step protocol, also available on protocols.io. (PDF)

S1 Video Video tutorial of trans-cardiac neonatal perfusion. Part1.
(MP4)

S2 Video Video tutorial of trans-cardiac neonatal perfusion. Part2.
(MP4)
