# Supplementary material for: Trans-cardiac perfusion of neonatal mice and immunofluorescence of the whole body as a method to study nervous system development
Source: PLoS One. 2022 Oct 13;17(10):e0275780. doi: 10.1371/journal.pone.0275780 (PMC9560478; doi:10.1371/journal.pone.0275780)
Supplement: S1 File — (PDF) [file pone.0275780.s001.pdf]

Aug 23, 2022

Trans-cardiac perfusion of neonatal mice and immunofluorescence of the whole body

Andrea Pérez Arévalo[1], Anne-Kathrin Lutz[1], Ekaterina Atanasova[1], Tobias M. Böckers[1,2]

[1]Institute for Anatomy and Cell Biology, Ulm University, Ulm, Germany;

[2]Deutsches Zentrum für Neurodegenerative Erkrankungen (DZNE), Ulm Site, Ulm, Germany

1 Works for me

Share

dx.doi.org/10.17504/protocols.io.bp2l61ow5vqe/v1

Anne-Kathrin Lutz

DISCLAIMER

DISCLAIMER – FOR INFORMATIONAL PURPOSES ONLY; USE AT YOUR OWN RISK

The protocol content here is for informational purposes only and does not constitute legal, medical, clinical, or safety advice, or otherwise; content added to protocols.io is not peer reviewed and may not have undergone a formal approval of any kind. Information presented in this protocol should not substitute for independent professional judgment, advice, diagnosis, or treatment. Any action you take or refrain from taking using or relying upon the information presented here is strictly at your own risk. You agree that neither the Company nor any of the authors, contributors, administrators, or anyone else associated with protocols.io, can be held responsible for your use of the information contained in or linked to this protocol or any of our Sites/Apps and Services.

Citation: Andrea PÃÂ©rez ArÃÂ©valo, Anne-Kathrin Lutz, Ekaterina Atanasova, Tobias M. BÃÂ¶ckers Trans-cardiac perfusion of neonatal mice and immunofluorescence of the whole body https://dx.doi.org/10.17504/protocols.io.bp2l61ow5vqe/v1

This is an open access protocol distributed under the terms of the **Creative Commons Attribution License** (https://creativecommons.org/licenses/by/4.0/), which permits unrestricted use, distribution, and reproduction in any medium,

ABSTRACT

Whole animal perfusion is a well-established method that has been used for the past decades in multiple research fields. Particularly, it has been very important for the study of the brain. The rapid and uniform fixation of tissue is essential for the preservation of its integrity and the study of complex structures. For small tissue pieces submerging in formaldehyde solution oftentimes is sufficient to get a good fixation, larger tissues or organs with a more complicated structure present a greater difficulty. Here, we report the precise parameters to successfully perform trans-cardiac perfusion of neonatal mouse pups that allows a uniform fixation of the whole body for subsequent structural analysis and immunohistochemistry. In comparison to standard perfusion procedures of adult mice, changes in the pump velocity, the buffer volume and in the needle size lead to high quality fixation of neonatal mice pups. Further, we present a whole-body section staining, which results in a highly specific immunofluorescence signal suited for detailed analysis of multiple tissues or systems at the same time. Thus, our protocol provides a reproducible and reliable method for neonatal perfusion and staining that can rapidly be applied in any laboratory. It allows a high quality analysis of cellular structures and expression profiles at early developmental stages.

ATTACHMENTS

Figure 1.pdf

DOI

dx.doi.org/10.17504/protocols.io.bp2l61ow5vqe/v1

PROTOCOL CITATION

Andrea Pérez Arévalo, Anne-Kathrin Lutz, Ekaterina Atanasova, Tobias M. Böckers 2022. Trans-cardiac perfusion of neonatal mice and immunofluorescence of the whole body. **protocols.io** https://protocols.io/view/trans-cardiac-perfusion-of-neonatal-mice-and-immun-cfmctk2w

FUNDERS ACKNOWLEDGEMENT

AIMS-2-TRIALS
Grant ID: 777394

DFG
Grant ID: 251293561

KEYWORDS

neonatal, protocol, trans-cardiac perfusion, immunohistochemistry, development

Citation: Andrea PÃÂ©rez ArÃÂ©valo, Anne-Kathrin Lutz, Ekaterina Atanasova, Tobias M. BÃÂ¶ckers Trans-cardiac perfusion of neonatal mice and immunofluorescence of the whole body https://dx.doi.org/10.17504/protocols.io.bp2l61ow5vqe/v1

This is an open access protocol distributed under the terms of the **Creative Commons Attribution License** (https://creativecommons.org/licenses/by/4.0/), which permits unrestricted use, distribution, and reproduction in any medium,

LICENSE

This is an open access protocol distributed under the terms of the Creative Commons Attribution License, which permits unrestricted use, distribution, and reproduction in any medium, provided the original author and source are credited

IMAGE ATTRIBUTION

Figure 1. Schematic view of the protocol. a. Comparison between fixation by immersion and trans-cardiac perfusion in neonatal mice. The liver (L) is shown in detail. b. Set-up for the perfusion with the materials needed (1. Microscissors, 2. Straight Forceps, 3. Curved Forceps, 4. Small Scissors). c. Scheme of the surgical procedure during the perfusion. d. Scheme of the post-fixation procedure to prepare the tissue for cryosectioning.

CREATED

Aug 22, 2022

LAST MODIFIED

Aug 23, 2022

PROTOCOL INTEGER ID

68996

DISCLAIMER:

DISCLAIMER – FOR INFORMATIONAL PURPOSES ONLY; USE AT YOUR OWN RISK

The protocol content here is for informational purposes only and does not constitute legal, medical, clinical, or safety advice, or otherwise; content added to protocols.io is not peer reviewed and may not have undergone a formal approval of any kind. Information presented in this protocol should not substitute for independent professional judgment, advice, diagnosis, or treatment. Any action you take or refrain from taking using or relying upon the information presented here is strictly at your own risk. You agree that neither the Company nor any of the authors, contributors, administrators, or anyone else associated with protocols.io, can be held responsible for your use of the information contained in or linked to this protocol or any of our Sites/Apps and Services.

Preparation of solutions and material 2h

1 Prepare 20mL of formaldehyde solution by dissolving 4% w/v Paraformaldehyde (PFA, Sigma-Aldrich, 15127-500G) in Phosphate Buffered Saline without calcium and magnesium (PBS-/-, Gibco, 14190250). Stir until the powder is dissolved. Adjust the pH to 7.4 and filter the solution using Whatman® prepleated qualitative filter paper, Grade 598 ½ (Merck Millipore, WHA10312247). Keep the solution at 4°C.

Citation: Andrea PÃÂ©rez ArÃÂ©valo, Anne-Kathrin Lutz, Ekaterina Atanasova, Tobias M. BÃÂ¶ckers Trans-cardiac perfusion of neonatal mice and immunofluorescence of the whole body https://dx.doi.org/10.17504/protocols.io.bp2l61ow5vqe/v1

This is an open access protocol distributed under the terms of the **Creative Commons Attribution License** (https://creativecommons.org/licenses/by/4.0/), which permits unrestricted use, distribution, and reproduction in any medium,

2 Cool 20mL PBS-/- for each animal.

3 Prepare a solution of Xylacine (Rompun 2%, Bayer, 25mL) + Ketamine (Ketamin 10%, WDT) (520mg/Kg ketamine and 78mg/Kg Xylacine) in a saline solution (Sodium Chloride 0.9%, Braun, 3570380) to a final volume of 20uL per animal.

4 Prepare 10%, 20% and 30% sucrose (Sigma-Aldrich, S0389-500G) solution in PBS-/- (10mL per animal).

5 Prepare gelatin (Sigma, G1890-100G) solution (2.5g in 33.3mL of 10% sucrose solution) (10mL per animal).

6 Prepare blocking solution with 5% Fetal Bovine Serum (FBS, Gibco, 10500-064, Lot 08F3482K) in Phosphate Buffered Saline with Ca2+ and Mg2+ (PBS+/+, Gibco, 14040141).

7 Prepare DAPI (Roth, 6335.1) solution 1:50.000 in PBS-/-.

8 Preparation of the pump.
a.Place two 50ml falcon tubes on ice, one with formaldehyde solution and the other one with PBS-/-, both at 4°C, and introduce one of the ends of the perfusion pump tube into the PBS-/- filled falcon.
b.Clean the pump system with PBS-/- at 4°C, kept on ice, until all air bubbles are eliminated.
c.Fill the system with PBS-/- at 4°C.
d.Attach a 27G needle (Henke Sass Wolf, 4710004525) to the other end of the plastic tube (0.3mm diameter).
e.Set the perfusion pump (Ismatec, GZ-78016-02) speed to 1ml/min.

9 Preparation for surgery.
Prepare the tools by sterilizing them.
1.Micro scissors (Micro Vannas, Straight, Sharp/Sharp, 8cm, No.15000-00)
2.Scissors (Extra Fine Bonn, Straight, Sharp/Sharp, 8.5cm, No. 14084-08)
3.Straight forceps (Moria MC40, Straight, 11cm, No. 11370-40)
4.Curved forceps (Dumont #7 Fine tips, 11cm, No. 11274-20)

Procedure

10 Animal fixation and perfusion.

Citation: Andrea PÃÂ©rez ArÃÂ©valo, Anne-Kathrin Lutz, Ekaterina Atanasova, Tobias M. BÃÂ¶ckers Trans-cardiac perfusion of neonatal mice and immunofluorescence of the whole body https://dx.doi.org/10.17504/protocols.io.bp2l61ow5vqe/v1

This is an open access protocol distributed under the terms of the **Creative Commons Attribution License** (https://creativecommons.org/licenses/by/4.0/), which permits unrestricted use, distribution, and reproduction in any medium,

I.Immobilize the pup and inject 20ul of the solution prepared previously to the animal in the intraperitoneal area using an insulin syringe (Insulin syringes with needle (BD Medical, 324826)).
II.Wait until the pup has no reflexes when you pinch the paw or the tail.
III.Fix the animal, using a small needle on each extremity, onto the perfusion platform.
IV.Grab the skin in the center of the abdomen and cut a small incision using the micro scissors (Figure 1C).
V.Continue separating the skin from the thorax of the animal and cutting up on both sides (Figure 1C).
VI.Grab onto the end of the sternum, make a small incision and cut on the same level on both sides, opening the thoracic cavity.
VII.Cut the diaphragm carefully without damaging the heart (Figure 1C).
VIII.Cut the ribs on both sides and on top to make the heart available for manipulation.
IX.With the micro scissors cut the right atrium to let the blood out of the circulatory system.
X.Insert the tip of the 27Gneedle into the left ventricle, being careful of not passing through the middle wall and into the right ventricle.
XI.Once you are sure you are in the correct position, start the pump.
XII.Clean the circulatory system letting 10mL of PBS-/- run through. The liver will change into a pale color (Figure 1C).
XIII.Once the blood is out, change the solution to formaldehyde solution. Turn off the pump to make sure no bubbles enter the system and change the tube from the falcon with PBS-/- to the one with formaldehyde solution.
XIV.Once the tube is changed you can turn on the pump again.
XV.Let run through 10mL of formaldehyde solution. You can check if the perfusion is working by examining the stiffness of the neck in the pup.
XVI.Once the animal is perfused, turn off the pump and continue with the extraction of your desired tissue. Alternatively, directly submerging the whole body into formaldehyde solution for post-fixation.

11 Post fixation.
I.Submerge your tissue of interest or the whole body in a falcon with formaldehyde solution at 4°C overnight. This time can be cut down depending on the size of your tissue (whole body: overnight, brain: 1.5-2 hours.)
II.Afterwards, wash away the formaldehyde solution using PBS-/-.
III.Then, submerge the tissue into 10% sucrose and keep it at 4°C until the tissue goes down to the bottom of the falcon.
IV.Increase the percentage of sucrose to 20% and repeat the last step. Then change it into 30% until the tissue goes down to the bottom of the falcon.

12 Embedding of tissue using gelatin.
I.Prepare the gelatin by heating it up to around 40°C.
II.Place the animal in a new 15mL falcon tube and cover it with 5ml of warm gelatin solution and keep it at 37°C for 15min.
III.In the meantime, prepare the embedding molds by adding a small bed of gelatin (around 2ml) and placing it in the fridge to allow it to set.
IV.Once the gelatin solidifies, place the embedding mold on top of a block of dry ice.

Citation: Andrea PÃÂ©rez ArÃÂ©valo, Anne-Kathrin Lutz, Ekaterina Atanasova, Tobias M. BÃÂ¶ckers Trans-cardiac perfusion of neonatal mice and immunofluorescence of the whole body https://dx.doi.org/10.17504/protocols.io.bp2l61ow5vqe/v1

This is an open access protocol distributed under the terms of the **Creative Commons Attribution License** (https://creativecommons.org/licenses/by/4.0/), which permits unrestricted use, distribution, and reproduction in any medium,

V.Before the gelatin in the mold freezes, place the pup in the intended position and hold it while adding 5ml of warm gelatin on top, until covered.
VI.Once the block is completely frozen, place at -80°C for short or long term storage.

13 Alternative embedding of tissue using O.C.T. (not recommended).
I.Place an embedding mold on top of a block of dry ice.
II.Fill up ¼ of the mold with OCT making a small bed to place the animal.
III.Let the OCT start to freeze, so when you place the animal it does not sink to the bottom.
IV.Once the OCT is halfway frozen, place the animal in the position desired.
V.Cover the animal and fill up the rest of the mold with more OCT making sure there are no bubbles.
VI.Let the OCT freeze completely and store the sample at -80°C.

14 Cryosectioning of tissue.
I.Put the block with the tissue at a -20°C freezer the day before the cryosectioning.
II.The next day, prepare the cryostat (Leica CM3050 S), inserting the blade (Feather Microtome Blades, C35 Type, 02.075.00.003) and adapting the temperature based on your protocol (-20°C chamber, -17°C arm, for cutting the whole body).
III.Place the block with the tissue inside the cryostat and keep it there for 30 min to allow it to adapt to the cryostat temperature.
IV.Cut the embedding mold and adhere the gelatin block to the insert using Tissue Tek O.C.T. (Sakura, 4583). Place the insert in the cryostat arm.
V.Trim away the excess gelatin, using the trimming feature.
VI.Once you can visualize the tissue, start cutting sections of 30µm and place them on a cryostat slide (SuperFrost Plus, VWR, 631-0108).
VII.Place the sections in a storage box and keep them at -20°C while cutting.
VIII.Keep the box with the sections at -80°C for long term storage.

Immunohistochemistry

15 -Take out the slides with the samples that were cut and kept at -80°C.
-Leave them to dry at RT, and once the glass is dry surround the sample with a small layer of ImmEdge Hydrophobic Barrier PAP Pen (Vector, H-400).
-Wait until the hydrophobic liquid is dry.
-Block the samples using 500uL of blocking medium (5% FBS in PBS+/+ plus 0.3% Triton 100x (Merck Millipore, 1122980101)) for 4 hours at RT.
-After that, add the primary antibodies diluted in the blocking medium.
-Iba1 1:500(Synaptic Systems, 234 004)
-GFAP 1:500 (Agilent Dako, Z033429-2)
-α-Actinin 1:500 (Sigma-Aldrich, A7811)
-Incubate the primary antibodies for 48 hours at 4°C.
-Then, wash four times with PBS+/+. First a quick wash, and then a 10 minute, 20 minutes and 30 minutes wash, at RT.
-Next, add the secondary antibodies diluted in blocking medium, and incubate for 2 hours at RT.
-AlexaFluor 488 Guinea Pig 1:500 (706-545-148) (Jackson Immuno Research)
-AlexaFluor 594 Rabbit 1:500 (711-585-152) (Jackson Immuno Research)

Citation: Andrea PÃÂ©rez ArÃÂ©valo, Anne-Kathrin Lutz, Ekaterina Atanasova, Tobias M. BÃÂ¶ckers Trans-cardiac perfusion of neonatal mice and immunofluorescence of the whole body https://dx.doi.org/10.17504/protocols.io.bp2l61ow5vqe/v1

This is an open access protocol distributed under the terms of the Creative Commons Attribution License (https://creativecommons.org/licenses/by/4.0/), which permits unrestricted use, distribution, and reproduction in any medium,

-AlexaFluor 647 Mouse 1:500 (715-695-151) (Jackson Immuno Research)
-After the secondary antibodies incubation, wash again with PBS-/- two times, one quick and 10 minutes.
-With the third wash, add the DAPI antibody, and incubate for 15 minutes at RT.
-Dapi 1:50.000 (Roth, 6335.1)
-Wash again with PBS-/- for 35 minutes at RT.
-Take away the wash, and leave the samples to dry a little bit.
-Mount the sections using VectaMount Permanent Mounting Medium (Biozol, VEC-H-5000)and a glass coverslip (VWR, 24x50mm, 631-1574).
-Image the samples using a confocal microscope (Leica DMi8 Confocal Microscope) and software (Leica software).

Citation: Andrea PÃÂ©rez ArÃÂ©valo, Anne-Kathrin Lutz, Ekaterina Atanasova, Tobias M. BÃÂ¶ckers Trans-cardiac perfusion of neonatal mice and immunofluorescence of the whole body https://dx.doi.org/10.17504/protocols.io.bp2l61ow5vqe/v1

This is an open access protocol distributed under the terms of the **Creative Commons Attribution License** (https://creativecommons.org/licenses/by/4.0/), which permits unrestricted use, distribution, and reproduction in any medium,
